# Development of Benchmark Curves to Early Detect Health-Related Productivity Deviations Using Production Indicators in Swine Nursery and Finishing Lots

**DOI:** 10.1155/tbed/9952020

**Published:** 2025-08-13

**Authors:** Mafalda Pedro Mil-Homens, Ximena Paz Portal, Edison Magalhaes, Tyler Holck, Joel Stave, Jordan Gebhardt, Eduardo Costa, Derald Holtkamp, Fernanda Dórea, Chong Wang, Giovani Trevisan, Daniel Linhares, Gustavo Silva

**Affiliations:** ^1^Department of Veterinary Diagnostic and Production Animal Medicine, College of Veterinary Medicine, Iowa State University, Ames, Iowa, USA; ^2^Department of Animal Health and Anatomy, Faculty of Veterinary Medicine, Autonomous University of Barcelona, Barcelona, Spain; ^3^Hanor Company, Enid, Oklahoma, USA; ^4^Department of Animal Science, College of Agriculture and Life Science, Iowa State University, Ames, Iowa, USA; ^5^Feed His People, LLC, Gilbert, Iowa, USA; ^6^Prairie Systems, Spencer, Iowa, USA; ^7^Department of Diagnostic Medicine/Pathobiology, College of Veterinary Medicine, Kansas State University, Manhattan, Kansas, USA; ^8^Department of Epidemiology, Bio-informatics, and Animal Models, Wageningen University, Wageningen, Netherlands; ^9^Swedish Veterinary Agency, Uppsala, Sweden; ^10^Food and Agriculture Organization of the United Nations, FAO, Rome, Italy; ^11^Department of Statistics, College of Liberal Arts and Sciences, Iowa State University, Ames, Iowa, USA

**Keywords:** Bayesian statistics, benchmarking, nursery and finishing, resampling techniques, surveillance, swine

## Abstract

The nursery and finishing phases are critical for profitability and sustainability in swine production, but effective methods for early disease detection in these stages remain underdeveloped. This study used routinely collected production indicators from nursery (42–56 days postfarrowing) and finishing lots (115–120 days postfarrowing) to create production benchmark curves for anomaly detection. These curves were developed for farms without diagnosed health challenges and compared to those with diagnosed health issues, based on tissue submissions and diagnostic codes (Dx codes). Statistical methods such as resampling techniques, Bayesian statistics, and standard deviation (SD) thresholds were employed to build the benchmark curves. The main objective was to test and compare the benchmarks using detection, early detection rate (EDR), time-to-detect (TTD), and false positive rate (FPR). Results showed that bootstrapping (BOOT), jackknife (JK), and Markov chain Monte Carlo (MCMC) methods provided the highest EDRs, although they were prone to false positives. For nursery lots, it was observed that using cumulative average with one SD for feed disappearance (EDR 49.2% and FPR 9.8%) and estimated weight (EDR 47.2% and FPR 8.8%) showed the best balance between EDR and FPR, and using MCMC for mortality showed the best balance between EDR and FPR (EDR 38.8% and FPR 13.8%). For finishing lots, using cumulative average with one SD showed a more balanced performance with FPR below 14.0% and EDR of 21.3% for feed disappearance, 67.4% weight, and 59.7% for mortality. These findings demonstrate the potential of using production indicators for early health challenge detection in swine operations.

## 1. Introduction

The swine industry in the United States is primarily structured around a multisite production system, where operations are divided into specialized site categories [1]. In the postweaning phase, pigs can be moved to nurseries and later to finishing sites. Nurseries are facilities where pigs generally remain from the postweaning stage until they reach around 56 days of age. Afterward, pigs are transferred to finishing sites, where they stay until reaching market weight, usually around 120 days of age, before being sold [2].

Maintaining herd health and maximizing productivity, especially during the nursery and finishing phases, is challenging [3, 4]. In these phases, productivity is focused on maximizing growth, feed efficiency, and reducing mortality [4–6]. As such, monitoring key indicators such as weight gain, feed intake, and mortality rates is essential for assessing the herd's health status, as deviations in these factors can signal the introduction of pathogens [7]. Currently, disease surveillance during the nursery and finishing phases primarily involve observing clinical signs and conducting diagnostic testing of biological samples as needed [8–10]. However, relying solely on farm staff to determine the need for diagnostics when clinical signs are present is a challenge for early outbreak detection [11, 12]. In contrast, implementing surveillance systems that also rely on production indicators can lead to the early detection of pathogen introductions in the herd [13–15], allowing swine producers to take proactive measures such as implementing diagnostic investigations, optimizing medication regimens to reduce clinical and production impacts, adjusting animal flow, and enhancing biosecurity measures between interconnected sites (e.g., employees, trucks, supplies, and maintenance). These interventions can minimize disease impact and prevent further spread across other farms [14, 16, 17].

Monitoring production indicators to identify cases before confirmed diagnosis is a key aspect of the shift in disease surveillance [16]. The core of this approach in disease surveillance is to use a proactive approach where production indicators are monitored continuously to detect potential disease outbreaks. Benchmark curves, as statistical tools to establish reference points for normal production performance, play an essential role in this context. These curves can be applied both for within-farm comparisons over time, to identify deviations relative to a farm's own stable production history, and for between-farm comparisons, to contrast farms or systems with stable conditions against those experiencing disruptions [18, 19]. However, focusing on within-farm temporal deviations helps to minimize the confounding effects of between-farm variability, which can mask the impact of mild or emerging outbreaks [20]. Production indicators such as growth rates or feed efficiency can thus be effectively monitored to detect early signs of health challenges [18, 19]. The systematic monitoring of production indicators, aided by benchmark curves, enables the early identification of deviations from normal productivity, serving as triggers for thorough investigations and allowing for a more proactive response to potential challenges. The change for monitoring available production indicators data can help reduce the need for continuous, resource-intensive diagnostics, while still allowing for the prompt identification and resolution of emerging issues, ultimately supporting a more efficient and targeted farm management strategy [14–16, 20].

Currently, there is a knowledge gap regarding the use of production indicators in the nursery and finishing phases for effective syndromic surveillance systems. This swine production phase often lacks clear benchmarks and diagnostic tools to effectively address performance variations [19]. The challenges in accurately tracking deviations in production indicators, coupled with limited understanding of the methods that can be used to detect these deviations, underscore the need for more integrated data systems and benchmarking methods to enhance the detection of deviations in productivity. Leveraging routinely collected farm data could provide valuable insights to support decision-makers in the field. Numerous studies have demonstrated the efficacy of syndromic surveillance in swine breeding herds [14, 15]. For instance, applying statistical process control (SPC) charts to monitor productivity indicators has facilitated the early detection of deviations linked to porcine reproductive and respiratory syndrome virus (PRRSV) outbreaks, often preceding the identification of an outbreak by results of diagnostic testing and test results [14, 15]. Additionally, cumulative sum control charts have been employed to identify alarming trends in mortality rates among sows and piglets [21]. SPC charts offer a systematic approach to identifying variations indicative of potential anomalies, although they assume that the process under investigation is stable over time. This constraint can hinder their effectiveness in dynamic environments [22]. In contrast, Bayesian analysis offers a flexible and robust alternative for anomaly detection, particularly in dynamic environments where traditional methods may fall short. By integrating prior knowledge and continuously updating beliefs with incoming data, Bayesian methods can adapt to fluctuating conditions and provide probabilistic assessments of anomalies [22–24]. This adaptability makes it an ideal choice for monitoring production indicators in real-time, where conditions may change rapidly, and unexpected variations can occur. Nonparametric resampling techniques, such as bootstrapping (BOOT) and jackknife (JK) [25–28], complement Bayesian methods by enhancing the reliability of benchmark curves, especially when there are limited real-time measurements or when parametric assumptions cannot be made [29]. These techniques generate multiple new sample sets, which refine parameter estimates and improve accuracy when traditional statistical methods may not be applicable [30]. Together, these methods provide a more robust framework for anomaly detection, as they allow for continuous learning and adaptation, ensuring more reliable and timely decision-making in complex production environments.

Continuous surveillance using routine production data at nursery and finishing sites offer significant advantages for swine health management, primarily because it enables the early detection of deviations from normal production patterns. By closely monitoring key production indicators, producers can quickly identify when performance metrics fall outside expected ranges, which provides an opportunity for early investigations and interventions before health issues escalate. This proactive approach minimizes the impact of potential health challenges, reducing both economic losses and the need for more intensive interventions later on. Therefore, this study aimed to take advantage of data routinely collected in swine farms and build, test, and compare benchmark curves using production indicators by considering early detection (percentage of deviations detected before the positive diagnostic result) and time-to-detect (TTD) a health challenge (time between the first deviation and the positive diagnostic result) during the nursery and finishing phases.

## 2. Materials and Methods

### 2.1. Overview of the Study

Benchmark curves for nursery and finishing lots were created by selecting lots without health challenges that had mortality rates within thresholds based on standard deviation (SD) and median absolute deviation (MAD). Lots exceeding these thresholds were excluded due to the high mortality, possibly indicating underlying undiagnosed health challenges that could affect the ideal benchmark curve. After selecting the appropriate lots, daily averages for each production indicator (estimated feed disappearance, estimated weight, and mortality) were calculated. To build the benchmark curves, various techniques were applied, including averaging with SD, applying a 95% confidence interval, and using parametric and nonparametric resampling methods. Once the benchmark curves were established, raw data from lots with known health challenges were compared to the benchmark curves. Early detection rate (EDR), late detection rate (LDR), and TTD, the health challenge, were calculated based on deviations in each production indicator from the benchmark curves. By taking advantage of underutilized data from swine nurseries and finishing lots, the authors aimed to provide decision-makers with a method that can be used routinely at the sites, aiding in anomaly detection.

### 2.2. Study Design

A retrospective study utilized daily data from 60 nursery farms (~56 days postweaning) between 2020 and 2022 and 193 finishing farms (~120 days postnursery) between 2021 and 2023 belonging to one swine production system from the United States Midwestern region, using the lot as the observational unit. The feed allocation system (FAS) from Prairie Systems (Spencer, Iowa), a comprehensive livestock management and feed ordering platform that connects truckers, mills, veterinarians, and growers, provided daily data that included daily farm inventory, daily cumulative mortality, daily cumulative estimated feed disappearance per head (kg), and daily cumulative estimated weight per head (kg).

The equations used to calculate the estimated feed disappearance and estimated weight are proprietary (FAS). However, the biological basis and the nonproprietary components included in these models are described here to ensure transparency. Feed disappearance was estimated using a combination of site-level production and logistical data. The model incorporated daily inventory per site, a standard average daily feed intake (ADFI) curve appropriate for the production phase (nursery or finishing), daily records of feed ordered and delivered, bin capacity, and the number of days on feed. This approach assumes that actual feed use is driven by both the population of animals on site and expected feed consumption patterns over time. The use of delivery records and standardized intake curves to model feed disappearance is consistent with common practices in commercial swine systems [31] and has been described in [32]. The estimation of average weight per head was based on daily inventory data, a standardized growth curve corresponding to the production phase, a feed conversion ratio (FCR) curve, daily feed delivery records, and the number of days on feed. This method reflects well-established relationships between feed intake, growth rate, and body weight in swine production [33, 34]. While the exact functional forms and coefficients are proprietary, the underlying logic is rooted in known biological principles and industry-standard performance modeling.

### 2.3. Health Challenge

It was the production system's policy to collect samples for diagnostics when clinical signs were present. Data concerning the health challenge diagnostic investigations using tissue submission and the sow farm weekly disease status for PRRSV and porcine epidemic diarrhea virus (PEDV) were provided by the swine production system. Once diagnostic results were finalized at the Iowa State University Veterinary Diagnostic Laboratory, diagnosticians assigned one or more standardized diagnostic codes (Dx codes) to the cases, documenting the identified pathogen and any associated lesions [35]. According to the diagnostician's findings from the tissue submissions, the pathogens were divided into four categories according to the pathologist's criteria: digestive, respiratory, multiple systems affected, and systemic (Table 1).

### 2.4. Selection of Benchmark Lots

**Figure 1:**
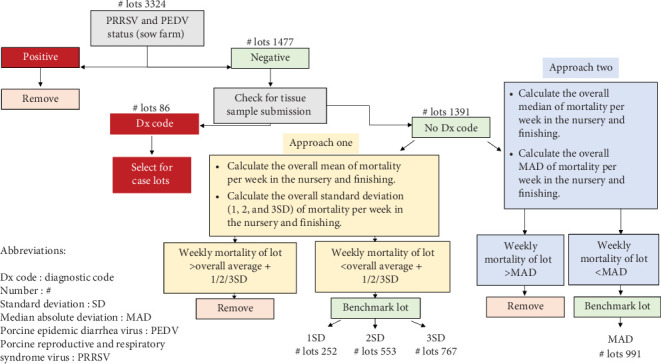
Lot selection diagram. Diagram with the different approaches used to select benchmarks and case lots of the studied nursery and finishing pig farms. A total of 1477 farms were considered for lot selection, with data between 2020 and 2023.

To select benchmark lots, the eligibility criteria were: (1) lots should originate from PEDV-negative sow farms and weaning negative pigs for PRRSV according to the American Association of Swine Veterinarians (AASV) classification [36]; (2) lots should not have any tissue submissions for any pathogen in the nursery and finishing phases. Additionally, the process of selecting benchmark lots involved two distinct stages. Initially, weekly mortality data were used to identify lots with consistent mortality patterns that would serve as a basis for further analysis. The selection criteria for these lots were based on two statistical methods, SD and MAD, which were applied to the weekly data to filter out lots with extreme mortality values (Figure 1). These two approaches provided an initial list of benchmark lots, which were then used to request daily data (feed, mortality, and weight) from the Prairie Systems company for further analysis. In the first approach, the overall mean and SD of mortality were determined, and lots with mortality below or equal to the mean plus one, two, and three SD were selected as benchmark lots. In the second approach, the overall median and MAD were calculated. The MAD is the median of absolute deviation from the data's median of mortality for a lot for a specific week. This method is preferred over the average because it deals better with non-normal distributions and outliers [37]. The lots with mortality below or equal to the median plus the MAD were selected as benchmark lots. The information concerning benchmark lots for the nursery and the finishing phases can be found in Table 2.

### 2.5. Selection of Health-Challenged Lots

To select the health-challenged lots or “case” lots, the eligibility criteria were that the lots should originate from sow farms negative for PEDV and weaning negative pigs for PRRSV (status of II, III, or IV according to the AASV classification [36]). Additionally, there should be reports of Dx code from tissue submissions that would represent a health challenge (Figure 1). The information concerning the case lots for the nursery and finishing phases can be observed in Table 3.

### 2.6. Selection of Thresholds

The unit of analysis in this study was the day in each phase (nursery and finishing). Benchmark curves were created by calculating daily cumulative averages for each production indicator across all benchmark lots. Different methods were used to build the curve and its thresholds. This daily curve served as a benchmark to compare the performance of lots experiencing health challenges (case lots). For case lots, the raw daily data for each indicator were compared directly to the benchmark curve, allowing for the identification of deviations and assessment of performance differences.

### 2.7. Average and Median

To build benchmark curves, different methods were tested. Different SDs (1, 2, and 3SD) and reference intervals (REF INT) (2.5^th^–97.5^th^ percentile) from the daily average of the cumulative mortality, cumulative estimated feed disappearance (kg), and cumulative estimated weight (kg) of all benchmark lots were also considered to create thresholds. The cumulative median and MAD were also used to create thresholds for the benchmark lots.

### 2.8. Nonparametric Resampling Techniques

Two resampling techniques were used to build the benchmark curves: BOOT and JK. BOOT is a resampling technique used to estimate the variability of survival estimates and derived statistics such as confidence intervals. It involves repeatedly sampling with replacement from the original dataset to create multiple bootstrap samples, from which survival curves are generated to assess uncertainty in the survival analysis results [38]. For this method, the number of bootstrap samples was set to 1000, and the BOOT for each row was implemented using the R package boot [39]. JK involves systematically leaving out one observation at a time from the original dataset to create multiple JK samples. In this approach, the number of JK samples was the same as the number of observations in the dataset.

### 2.9. Resampling Bayesian Statistics

Markov chain Monte Carlo (MCMC) models were tested to build the benchmark curves. One of the MCMC models used Stan, implementing Bayesian inference techniques, specifically Hamiltonian Monte Carlo (HMC) and its variant, the No-U-Turn (NUT) sampler, to sample from the posterior distribution of the model (MCMC HMC NUT). This method effectively explores the parameter space by utilizing gradient information, which enhances convergence and reduces autocorrelation among samples. The Stan model was defined to analyze daily mortality, which was assumed to follow a binomial distribution, and feed disappearance and weight, which were assumed to follow a gamma distribution. The other MCMC model used JAGS, which employs Bayesian inference through Gibbs sampling and Metropolis-Hastings (MCMC MH) sampling to estimate parameters in a model that also assumes a binomial distribution for mortality and a gamma distribution for feed disappearance and weight. Both methods used three chains; the MCMC MH method employed 2000 iterations, while the MCMC HMC with NUT sampler utilized 5000 iterations. To assess convergence, the Gelman–Rubin diagnostic (*R*-hat) was examined, which evaluates the consistency between multiple chains by comparing the within-chain and between-chain variances [40]. Convergence is indicated when *R*-hat values are below 1.1, with values greater than 1.1 suggesting that additional iterations may be required [41]. For both MCMC MH and MCMC HMC NUT, the *R*-hat values were below 1.1, indicating convergence.

### 2.10. Performance of the Benchmark Curves to Detect a Deviation

**Figure 2:**
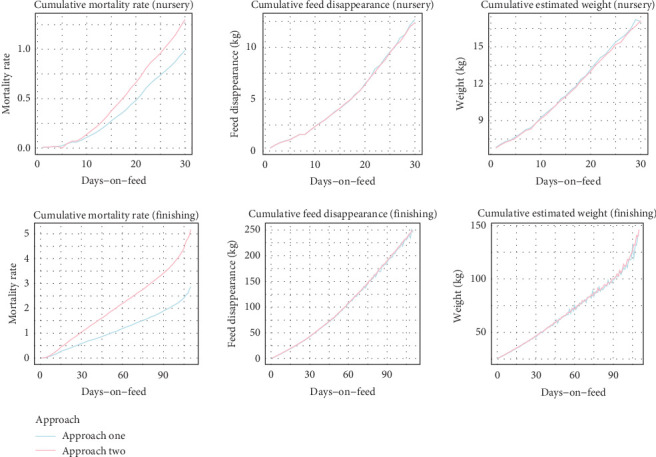
Benchmark curves. Line charts of the benchmark curves for the nursery (top three charts) and finishing (bottom three charts) phases. Approach one, using 3SD for the selection method for the benchmark lots, can be observed in blue, and approach two can be observed in pink. Bootstrapping was used to build the benchmark curve.

**Figure 3:**
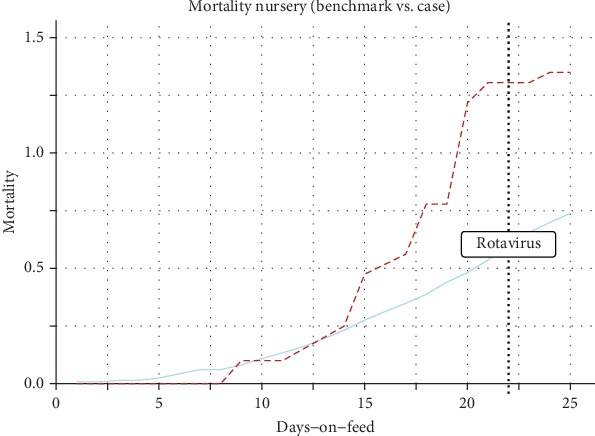
Early detection case example. Line chart of the mortality benchmark curve (blue line) and positive rotavirus cases (red dotted line) for the nursery phase. Approach one, using 3SD, was used for the selection method for the benchmark lots. Bootstrapping was used to build the benchmark curve.

**Figure 4:**
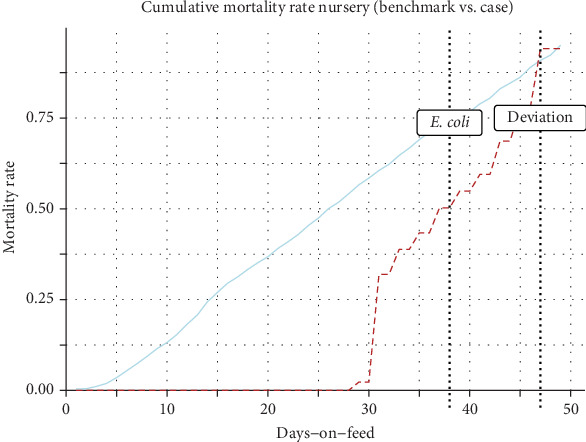
Late detection case example. Line chart of the mortality benchmark curve (blue line) and positive hemolytic *Escherichia coli* case (red dotted line) for the nursery phase. Approach one, using 3SD, was used for the selection method for the benchmark lots. Bootstrapping was used to build the benchmark curve.

Benchmark curves were created (Figure 2), and the case lots' indicators were individually juxtaposed with the benchmark curves to evaluate deviations. Deviations referred to the first point at which the case curve exceeded the benchmark threshold.  Figure 3 presents a case involving a Rotavirus outbreak, which demonstrates early detection, with deviations appearing prior to diagnostic confirmation. In contrast, Figure 4 depicts a hemolytic *Escherichia coli* case where detection occurred later, after clinical confirmation, highlighting a scenario where the surveillance system was less sensitive. The study computed (EDRs) (number of lots with deviations one to 10 days before diagnostic evidence report over the total number of reports), DR (number of lots with deviations on the day of the diagnostic evidence report over the total number of reports), LDR (lots with deviations one to 5 days after the diagnostic evidence report, over the total number of reports), TTD a health challenge (number of days between the day with the first deviation until the day with the diagnostic results), and false positive rate (FPR) (false positives in benchmark lots or nonhealth challenged lots divided by the sum of false positives in nonhealth challenged lots and true negatives in nonhealth-challenged lots) (Table 4). The methods that showed the highest DR, EDR, TTD, and lowest FPR were considered the best methods to assess performance. The analysis was done in the statistical programming environment R Statistical Software (v4.4.0; R Core Team 2024) and Microsoft Excel.

## 3. Results

### 3.1. Nursery

#### 3.1.1. Descriptive

In the nursery phase, 25 lots faced digestive and respiratory health challenges, with lots having multiple systems affected.

#### 3.1.2. Approach One

For approach one, the highest EDR for all indicators used MCMC MH and MCMC HMC NUT, although the FPR was above 30.0% for all indicators. Therefore, the methods that showed a better balance between EDR and FPR used cumulative average with 1SD for feed disappearance (EDR 49.2%, DR 56.0%, LDR 45.6%, TTD 5.4 days, and FPR 9.8%), using MCMC MH for mortality (EDR 38.8%, DR 44.0%, LDR 51.8%, TTD 4.7 days, and FPR 13.8%), and using cumulative average with 1SD for estimated weight (EDR 47.2%, DR 24.0%, LDR 23.2%, TTD 5.9 days and FPR 8.8%) (Table 5).

#### 3.1.3. Health Challenge for Approach One

Cases associated with multiple systems affected had the highest EDR for feed disappearance (48.0%) and weight (48.0%) using MCMC HMC NUT. Cases associated with multiple systems affected had the highest EDR for mortality (19.6%) using BOOT and JK (Supporting Information).

#### 3.1.4. Approach Two

For approach two, the method with the highest EDR for feed disappearance was BOOT with an EDR of 60.4%, although the FPR was 42.0%; therefore, the method that had a better balance between EDR and FPR using 1SD (EDR 48.0%, DR 48.0%, LDR 45.6%, TTD 5.4 days, FPR 9.2%). Concerning mortality, the method with the highest performance was the MCMC MH, with EDR of 40.0%, DR of 60.0%, LDR of 57.6%, TTD of 4.7 days, and FPR of 10.8%. Lastly, for weight, the models with the highest EDR were BOOT, JK, and MCMC HMC NUT, with EDR of 22.0%, DR of 40.0%, LDR of 37.6%, TTD of 4.7 days, and FPR approximately 52% (Table 5).

#### 3.1.5. Health Challenge for Approach Two

Cases associated with digestive health challenges had the highest EDR for feed disappearance (34.6%) using BOOT. Cases associated with multiple systems affected had the highest EDR for weight (12.8%) using BOOT, JK, and MCMC HMC NUT. Cases associated with multiple systems affected had the highest EDR for mortality (20.4%) using MCMC MH (Supporting Information).

### 3.2. Finishing

#### 3.2.1. Descriptive

There were 61 lots with health challenges in the finishing phase, with lots facing digestive, respiratory, and systemic health challenges, with lots also having multiple systems affected.

#### 3.2.2. Approach One

For approach one, the highest EDR for feed disappearance and estimated weight used the threshold method BOOT for feed disappearance (EDR of 39.7%) and for the estimated weight (EDR of 68.9%), although for both, the FPR was above 57.0%. Using a cumulative average with 1SD for feed disappearance (EDR 21.3%, DR 23.0%, LDR 29.8%, TTD 5.4 days, and FPR 6.3%) showed a more balanced performance, and using a cumulative average with 1SD for the estimated weight (EDR 67.4%, DR 70.5%, LDR 67.2%, TTD 5.3 days, and FPR 13.5%) also showed a more balanced performance. For mortality, using the cumulative average and 1SD as the threshold method (EDR 59.7%, DR 73.8%, LDR 77.1%, TTD 5.2 days, and FPR 12.0%) was the more balanced method in terms of performance, although the method that showed the highest EDR was MCMC MH with EDR of 61.6% (Table 6).

### 3.3. Health Challenge for Approach One

Cases associated with multiple systems affected had the highest DR for feed disappearance (27.9%), mortality (54.1%) using BOOT, JK, and MCMC MH, and weight (49.2%) using BOOT (Supporting Information).

### 3.4. Approach Two

For approach two, the methods with the highest EDR were BOOT, MCMC HMC NUT, and MCMC MH, although the FPR was above 35.05%. With that, the threshold method that showed a more balanced performance of EDR and FPR was using the cumulative average and 1SD for all production indicators. Feed disappearance showed an EDR of 38.7%, DR of 25.2%, LDR of 37.4%, TTD of 5.7 days, and FPR of 13.0%. Mortality showed an EDR of 29.2%, DR of 49.2%, LDR of 52.5%, TTD of 4.9%, and FPR of 12.6%, and estimated weight showed an EDR of 38.9%, DR of 39.3%, LDR of 32.8%, TTD of 5.4 days, and FPR of 11.8% (Table 6).

### 3.5. Health Challenge for Approach Two

Cases associated with multiple systems affected had the highest EDR for feed disappearance (37.1%) using BOOT, mortality (36.7%) using MCMC MH, and MCMC HMC NUT, and weight (24.6%) using MCMC MH (Supporting Information).

## 4. Discussion

Tracking production indicators at nursery and finishing swine sites is crucial for evaluating herd health and detecting potential pathogen introduction. Continuous monitoring allows producers to identify unusual changes in production that may signal the presence of a new infectious agent. By systematically collecting and analyzing data over time, trends in the data can be identified, facilitating timely interventions [42, 43]. Early recognition of symptoms leads to faster diagnosis, prompt treatment, and enhanced biosecurity measures to prevent further spread and minimize economic losses. Implementing such early warning systems not only helps maintain herd health but also promotes animal welfare, longevity, and overall productivity [14, 15, 20, 44].

Previous research has studied the value of monitoring production indicators to guide decision-making at various stages of swine production [21, 42, 43]. This study focused on creating production system-specific benchmark curves using production data to aid decision-makers in addressing a gap in the literature on implementing this methodology in the nursery and finishing lots. Although there are publications that focus on the same purpose, that is, to take advantage of routinely collected on-farm data to create a surveillance system. One approach used cumulative curves to detect deviations in water intake in sick calves [45], and other approaches used moving averages to detect deviations in production indicators, such as abortions, prenatal losses, and dead sows, to detect deviations associated with PRRSV outbreaks [14, 15, 20]. In this study, a Bayesian analytical framework and nonparametric resampling techniques were used. The first offers probabilistic anomaly detection that can flexibly incorporate prior knowledge and adapt to real-time data, which makes it more responsive to fluctuations than traditional threshold-based approaches [24], and the second is particularly useful in contexts with limited or non-normally distributed data, which often occurs in swine production systems [26]. However, despite working with different methods, all the studies showed that deviations connected to health challenges can be detected before an official diagnosis is confirmed.

Averages with SD, resampling techniques, and Bayesian statistics were the methods implemented in this study. The Bayesian statistics techniques showed higher EDR than the other methods in the nursery lots, with estimated feed disappearance and estimated weight reaching an EDR of 95.2% using the MCMC HMC NUT and mortality reaching an EDR of 40.4% using the MCMC MH. The resampling techniques reached higher EDR than the other techniques in the finishing phase for estimated feed disappearance and estimated weight (47.1% and 68.9%, respectively) using BOOT, and the Bayesian techniques showed higher EDR than the other techniques for mortality (61.6%) using the MCMC MH method. Despite the high EDR, these methods also showed high FPR, which may be attributed to their resampling approach, which effectively captures variability in the data and increases sensitivity [46]. Additionally, the Bayesian methods can be prone to false positives because the MCMC will base its posterior distribution on the priors. The posterior thresholds might be too restricted if the prior distribution has low values, but one benefit of using these methods is their ability to handle missing data [47]. Conversely, threshold methods employing daily cumulative averages with 2SD and 3SD showed low DRs, highlighting their inadequacy in detecting deviations. This suggests that for timely and accurate disease surveillance, methods like BOOT, JK, or MCMC are more effective in detecting deviations early, although they are prone to false positives. It is important to note that false positives should be carefully considered in the context of swine production systems. While the methods used in this study show the presence of deviations in production indicators, they do not account for all the possible factors influencing those indicators, such as environmental conditions, management practices, and undiagnosed diseases. As a result, what may appear as a false positive could potentially reflect underlying issues that have not yet been reported or detected by the farm, warranting further investigation.

Implementing continuous surveillance with routinely collected production data at nursery and finishing sites provides considerable advantages for managing swine health, especially in light of labor constraints. Relying solely on farm personnel to detect clinical signs across multiple sites can delay diagnostics and compromise the accuracy of disease detection. In contrast, tracking production data on farm or electronically allows for near-real-time or real-time monitoring, facilitating automated data collection, analysis, and reporting. With this type of system implemented, producers can quickly spot deviations, enabling early interventions and reducing health impacts. Developing benchmark curves to compare current performance with historical data facilitates the early identification of anomalies, leading to a more effective response. This proactive approach supports better decision-making and helps sustain herd productivity.

While the models used in this study do not explicitly account for correlations and dependencies within the data, it is recognized that production indicators exhibit temporal correlations within lots. These indicators are biologically and operationally linked over time, meaning that performance on a given day is often influenced by the preceding days [48]. Ignoring this serial dependence may lead to underestimated variability, narrower confidence intervals, and an increased risk of false-positive anomaly detection [49]. This study serves as an initial exploration to test the feasibility of using different methods for benchmarking production curves in nursery and finishing lots, given the limited literature on anomaly detection for these phases of the swine industry. Future studies should incorporate statistical models that better capture these dependencies, such as autoregressive models, time-series approaches, or hierarchical Bayesian frameworks [41, 50, 51].

This research utilized data sourced from widely used databases in the pig farming industry. By focusing on daily data, the study aimed to identify subtle changes and potential short-term patterns or irregularities. Although data collection is prone to human error, it is important to emphasize that the data employed in this research is considered the most detailed and broadly accepted depiction of the dynamics in nursery and finishing lots, mirroring the actual conditions of farm operations. In addition, this study worked with a production system that implemented diagnostic investigation as soon as the pigs started to show clinical signs. It was still a limitation not to have ongoing diagnostic tests that could provide more detail on the disease introduction at the farm. Despite all tissue submissions being directed to the Iowa State Veterinary Diagnostic Laboratory, along with the routine surveillance samples, the production system also sends routine surveillance samples to the Oklahoma Diagnostic Laboratory, which may result in some lots of pigs with positive diagnostics being overlooked. Another limitation was not addressing the pig flow. Differences in flows could affect data consistency and introduce potential biases, as pigs with different origins might be exposed to distinct conditions, different barn settings, or disease pressures.

The focus of this research was to provide a general benchmarking overview of performance curves for nursery and finishing lots in swine populations, as there is a lack of research in this area, and to provide the decision-makers with information on methods that they can use to implement benchmark curves specific to their production system. Thus, examining pig flow across different stages of production was not the primary focus, though it is an important aspect that could be explored in future studies. Understanding how variations in flow, different farm origins, and potential seasonal fluctuations might affect health and performance metrics would provide valuable insights for more precise benchmarking in the future. For future research, it would be valuable to implement the benchmark curves in several production systems, at both the farm and production system levels, and test them in real-time. While mortality was used as the primary benchmark criterion due to its consistent availability and relevance in field decision-making, incorporating a multi-indicator approach to refine the benchmark lots selection could bring a better representation of what the ideal benchmark lots should be. Additionally, it would be interesting to test other approaches, such as machine learning models, and compare the performance with the methods mentioned in this study.

## 5. Conclusion

In summary, the findings indicated that constructing benchmark curves with indicators typically gathered at nursery and finishing lots facilitated the early identification of disease-related symptoms. Moreover, analyzing such data enables decision-makers to recognize the potential onset of disease sooner, offering critical insights for timely intervention, planning for disease management and eradication, and preventing disease transmission to additional locations.

## Figures and Tables

**Table 1:** Health challenge for nursery and finishing phases.

Phase	Health challenge	Pathogens
Nursery	Digestive	Coccidia; Hemolytic *Escherichia coli; Rotavirus*
	Respiratory	Influenza A
	Multiple systems affected	Hemolytic *Escherichia coli; Glaesserela parasuis;* Influenza A*; Mycoplasma hyorhinis; Pasteurella multocida;* PEDV
	Systemic	No systemic cases

Finishing	Digestive	Hemolytic *Escherichia coli*
	Respiratory	*Pasterurella multocida*; PRRSV
	Multiple systems affected	*Actinobacillus pleuropneumoniae; Actinobacillus suis;* Influenza A; *Mycoplasma hyorhinis;* PRRSV
	Systemic	PRRSV

*Note:* Health challenges according to the clinical findings from the tissue submission. A total of 1477 lots from a single production system were considered for analysis, with 86 lots being considered as positive for at least one of the pathogens mentioned in the table. The tissue submissions were done following the presence of clinical signs, and diseases were assigned to multiple categories according to the pathologists' criteria.

**Table 2:** Benchmark lots performance metrics.

Phase	Approach	Lots	Number of lots	Average weight out per head (kg)	Average days on feed	Average feed intake per head (kg)	Average head in	Average mortality (%)
Nursery	1	Benchmark 1SD	143	25.4	42	28.1	4016	2.1
		Benchmark 2SD	245	25.4	43	28.1	3986	2.5
		Benchmark 3SD	281	25.4	43	28.1	3982	2.6
	2	Benchmark MAD	291	25.3	42	28.1	4005	3.0

Finishing	1	Benchmark 1SD	109	137.8	122	316.5	1200	1.8
		Benchmark 2SD	308	137.4	123	317.5	1185	2.9
		Benchmark 3SD	486	137.0	123	319.1	1175	3.5
	2	Benchmark MAD	700	137.0	123	321.1	1174	4.2

*Note:* Description of the swine performance metrics for benchmark lots using different approaches and production phases. The average weight out per head, average days on feed, average feed intake per head, average head in, and average mortality were calculated from the selected benchmark lots postselection.

**Table 3:** Case lots performance metrics.

Phase	Number of lots	Average weight out per head (kg)	Average days on feed	Average feed intake per head (kg)	Average head in	Average mortality (%)
Nursery	25	24.9	43	28.0	4528	5.0
Finishing	61	135.2	125	326.4	1168	8.0

*Note:* Description of the swine performance metrics for case lots and production phases. The average weight out per head, average days on feed, average feed intake per head, average head in, and average mortality were calculated from the selected benchmark lots postselection.

**Table 4:** Methods's performance.

Metric	Calculation
Early detection rate (EDR)	$\frac{\text{Deviation }\left(10\text{ to }1\right)\text{ days before report}}{\text{All reports}}\times 100$
Detection rate (DR)	$\frac{\text{Deviation on the day of report}}{\text{All reports}}\times 100$
Late detection rate (LDR)	$\frac{\text{Deviation }\left(10\text{ to }5\right)\text{days after report}}{\text{All reports}}\times 100$
Time-to-detect (TTD)	Number of days between the day with the first deviation and the day of the diagnostic result
False positive rate	$\frac{\text{False positives in benchmark lots}}{\text{False positives in benchmark lots}+\text{true negatives in benchmark lots}}\times 100\text{\% }$

*Note:* Metrics for evaluation of the benchmark curves methods' performance.

**Table 5:** Result of performance metrics for nursery.

Benchmark lot selection method	Benchmark curve threshold	Avg EDR feed (%)	Avg DR feed (%)	Avg LDR feed (%)	Avg TTD feed (days)	FPR feed (%)	Avg EDR mort. (%)	Avg DR mort. (%)	Avg LDR mort. (%)	Avg TTD mort. (days)	FPR mort. (%)	Avg EDR weight (%)	Avg DR weight (%)	Avg LDR weight (%)	Avg TTD weight (days)	FPR weight (%)
1SD	1SD	49.20	56.00	45.60	5.43	9.75	37.60	44.00	50.40	4.93	12.94	47.20	24.00	25.60	5.87	8.80
	2SD	22.80	24.00	23.20	5.53	3.96	22.80	32.00	38.40	4.40	3.79	20.80	12.00	11.20	6.13	3.28
	3SD	18.40	16.00	18.40	5.43	3.59	15.60	20.00	24.80	4.13	1.60	14.40	8.00	11.20	5.89	2.53
	MAD	8.00	8.00	8.80	5.09	27.18	44.00	64.00	62.40	4.71	23.53	16.40	12.00	15.20	5.55	30.18
	REF INT	5.60	0.00	0.00	0.00	0.02	25.60	32.00	32.00	4.36	2.90	8.80	4.00	0.80	3.14	3.19
	BOOT	62.40	64.00	59.20	5.49	43.55	36.80	44.00	50.40	5.11	25.93	72.40	72.00	64.80	5.49	54.47
	JK	62.00	68.00	59.20	5.50	43.54	37.20	36.00	38.40	5.11	25.91	72.80	72.00	66.40	5.48	54.03
	MCMC HMC NUT	59.60	64.00	52.00	5.57	56.95	36.80	44.00	50.40	4.65	13.75	73.20	76.00	72.80	5.52	80.33
	MCMC MH	59.60	64.00	57.60	5.40	41.04	38.80	44.00	52.80	4.65	13.75	53.20	32.00	24.80	5.88	36.35

2SD	1SD	48.40	52.00	45.60	5.40	9.69	22.80	40.00	34.40	4.42	10.03	47.20	24.00	20.80	5.91	8.31
	2SD	21.60	24.00	22.40	5.63	4.32	11.60	16.00	12.00	3.90	4.40	18.40	12.00	8.80	6.09	3.34
	3SD	18.00	20.00	16.80	5.33	4.07	2.40	8.00	5.60	2.67	1.89	14.00	8.00	6.40	5.77	2.59
	MAD	6.00	8.00	6.40	5.20	28.23	32.40	48.00	44.80	4.56	26.05	18.40	12.00	12.80	5.76	30.15
	REF INT	0.00	0.00	0.00	0.00	0.01	2.80	4.00	4.80	4.00	2.85	2.80	0.00	0.80	3.43	3.29
	BOOT	61.20	64.00	57.60	5.48	42.72	30.40	44.00	50.40	4.66	29.67	72.80	72.00	63.20	5.47	53.02
	JK	60.80	64.00	57.60	5.48	42.73	30.40	44.00	50.40	4.66	29.68	72.40	68.00	63.20	5.49	53.11
	MCMC HMC NUT	68.40	56.00	55.20	5.66	42.69	17.60	36.00	34.40	4.48	29.67	48.80	44.00	32.80	5.65	53.03
	MCMC MH	59.20	64.00	57.60	5.40	41.13	32.80	44.00	44.80	4.63	29.68	54.40	32.00	25.60	5.82	36.40

3SD	1SD	49.20	56.00	45.60	5.43	9.75	17.20	32.00	26.40	4.21	12.66	47.20	24.00	23.20	5.87	8.80
	2SD	22.80	24.00	23.20	5.53	3.96	10.00	16.00	10.40	3.60	3.51	20.80	12.00	8.80	6.13	3.28
	3SD	18.40	16.00	18.40	5.43	3.59	2.00	8.00	4.80	3.00	1.32	14.40	8.00	8.80	5.89	2.53
	MAD	8.80	8.00	8.00	5.09	27.18	42.40	60.00	54.40	4.82	26.93	17.60	16.00	13.60	5.55	30.18
	REF INT	0.00	0.00	0.00	0.00	0.02	13.60	20.00	18.40	4.62	2.76	2.80	0.00	0.80	3.14	3.19
	BOOT	62.40	64.00	59.20	5.49	43.55	27.60	44.00	44.80	4.65	29.89	72.40	72.00	64.80	5.49	54.47
	JK	62.00	68.00	59.20	5.50	43.54	27.60	44.00	44.80	4.65	29.91	72.80	72.00	66.40	5.48	54.03
	MCMC HMC NUT	95.20	100.00	93.60	5.44	43.41	32.80	48.00	46.40	4.63	30.93	95.20	100.00	93.60	5.44	53.86
	MCMC MH	59.60	64.00	57.60	5.40	41.04	40.40	44.00	51.20	5.19	30.95	53.20	32.00	24.80	5.88	36.35

MAD	1SD	48.00	48.00	45.60	5.36	9.21	22.40	40.00	37.60	4.02	10.84	1.60	0.00	0.00	8.25	8.27
	2SD	20.40	20.00	20.80	5.59	5.00	14.40	24.00	21.60	3.58	10.84	0.00	0.00	0.00	0.00	3.76
	3SD	15.20	16.00	16.80	5.21	4.78	8.00	16.00	16.00	3.70	10.84	0.00	0.00	0.00	0.00	3.00
	MAD	43.20	44.00	44.00	5.50	28.15	36.00	52.00	46.40	4.37	10.84	1.60	0.00	0.00	8.25	29.49
	REF INT	0.00	0.00	0.00	0.00	0.01	8.00	8.00	11.20	3.35	10.84	0.00	0.00	0.00	0.00	3.30
	BOOT	60.40	64.00	56.00	5.44	42.02	32.80	56.00	54.40	4.44	10.84	22.00	40.00	37.60	4.73	52.15
	JK	59.60	64.00	56.80	5.43	41.95	28.40	44.00	47.20	4.63	10.84	22.00	40.00	37.60	4.73	52.01
	MCMC HMC NUT	8.80	4.00	3.20	6.68	41.90	34.80	48.00	49.60	4.54	10.84	22.00	40.00	37.60	4.73	52.00
	MCMC MH	59.20	64.00	57.60	5.40	40.61	40.00	60.00	57.60	4.65	10.84	3.20	4.00	0.00	7.13	35.69

*Note:* Performance metrics for each benchmark selection method and each benchmark curve method for the nursery phase. MCMC HMC NUT, Markov chain Hamiltonian Monte Carlo. TTD, time-to-detect disease.

Abbreviations: Avg, average; BOOT, bootstrapping; DR, detection rate; EDR, early detection rate; JK, jackkinfe; LDR, late detection rate; MAD, median absolute deviation; MCMC MH, Markov chain Monte Carlo Metropolis-Hastings; Mort., mortality; REF INT, reference interval; SD, standard deviation.

**Table 6:** Result of performance metrics for finishing.

Benchmark lot selection method	Benchmark curve threshold	Avg EDR feed (%)	Avg DR feed (%)	Avg LDR feed (%)	Avg TTD feed (days)	FPR feed (%)	Avg EDR mort. (%)	Avg DR mort. (%)	Avg LDR mort. (%)	Avg TTD mort. (days)	FPR mort. (%)	Avg EDR weight (%)	Avg DR weight (%)	Avg LDR weight (%)	Avg TTD weight (days)	FPR weigth (%)
1SD	1SD	21.31	22.95	29.84	5.42	6.31	59.67	73.77	77.05	5.16	11.95	66.89	70.49	70.49	5.36	16.20
	2SD	14.92	14.75	15.41	5.47	0.95	41.31	59.02	66.89	4.98	3.15	30.98	32.79	32.79	5.34	0.37
	3SD	11.48	11.48	10.49	5.50	0.30	27.21	52.46	52.79	4.88	1.11	6.23	9.84	9.18	4.79	0.06
	MAD	15.90	19.67	19.34	5.38	29.78	48.85	67.21	70.82	5.04	24.33	14.10	11.48	11.48	5.95	27.41
	REF INT	5.41	0.00	0.33	7.50	3.71	33.11	42.62	53.11	4.85	2.75	9.02	1.64	1.64	5.25	6.37
	BOOT	35.25	36.07	42.62	5.63	37.89	58.52	70.49	76.39	5.17	43.80	68.03	72.13	72.46	5.32	56.20
	JK	37.21	32.79	42.30	5.66	36.51	61.48	73.77	79.02	5.17	43.79	67.70	70.49	70.82	5.35	56.32
	MCMC HMC NUT	36.23	36.07	41.97	5.66	36.49	58.52	70.49	76.72	5.04	57.42	67.87	70.49	71.48	5.35	56.31
	MCMC MH	12.95	14.75	14.10	5.34	4.57	58.52	70.49	76.72	5.04	57.57	31.80	29.51	22.30	5.38	6.56

2SD	1SD	21.48	22.95	30.82	5.45	12.95	44.43	63.93	67.54	5.04	13.05	67.38	70.49	67.21	5.33	13.50
	2SD	15.41	14.75	14.75	5.50	2.13	27.05	50.82	53.11	4.88	3.59	34.26	36.07	33.44	5.31	1.42
	3SD	9.84	9.84	9.84	5.50	0.52	18.20	36.07	39.67	4.53	1.03	6.56	9.84	10.82	4.73	0.00
	MAD	25.41	27.87	27.54	5.38	26.43	40.33	62.30	68.85	4.42	26.79	15.74	14.75	11.15	5.75	27.53
	REF INT	2.95	6.56	3.93	5.22	4.34	15.41	34.43	36.07	3.33	2.59	3.44	3.28	3.28	5.48	4.64
	BOOT	39.67	45.90	46.23	5.50	59.93	50.16	63.93	69.18	5.13	40.21	68.85	72.13	72.46	5.36	57.11
	JK	39.67	44.26	48.20	5.50	61.29	50.00	63.93	69.18	5.14	40.21	68.36	70.49	71.15	5.38	56.97
	MCMC HMC NUT	23.61	14.75	8.20	5.69	60.60	49.84	67.21	74.43	5.04	40.20	0.00	0.00	0.00	0.00	56.98
	MCMC MH	17.05	16.39	22.30	5.68	11.93	50.16	67.21	74.43	5.02	40.21	40.33	40.98	40.98	5.39	10.59

3SD	1SD	20.82	22.95	29.51	5.35	12.63	36.89	59.02	65.25	4.76	11.95	66.23	70.49	69.51	5.33	16.20
	2SD	14.92	14.75	15.41	5.47	1.84	22.62	39.34	40.98	4.64	3.15	30.98	32.79	32.79	5.34	0.37
	3SD	11.48	11.48	10.49	5.50	0.38	11.64	29.51	32.13	3.94	1.11	6.23	9.84	9.18	4.79	0.06
	MAD	29.51	36.07	36.72	5.34	29.53	50.16	68.85	71.48	5.04	24.33	15.08	9.84	11.80	5.95	27.41
	REF INT	4.10	4.92	4.26	5.08	3.66	29.18	52.46	53.44	4.85	2.75	1.97	1.64	1.64	5.25	6.37
	BOOT	35.57	29.51	42.95	5.57	60.51	42.62	62.30	67.54	4.91	43.81	67.38	72.13	72.13	5.30	56.20
	JK	37.21	32.79	42.30	5.66	64.77	42.62	62.30	67.54	4.91	43.79	67.70	70.49	70.82	5.35	56.32
	MCMC HMC NUT	9.84	9.84	9.84	5.50	62.39	49.84	67.21	74.75	5.04	43.77	1.64	3.28	2.62	3.90	56.20
	MCMC MH	12.95	14.75	14.10	5.34	8.00	61.64	75.41	80.33	5.17	43.80	31.80	29.51	22.62	5.38	6.59

MAD	1SD	28.69	26.23	37.38	5.66	13.02	29.18	49.18	52.46	4.85	12.60	38.85	39.34	32.79	5.43	11.79
	2SD	15.57	16.39	14.10	5.60	1.75	14.43	34.43	38.36	4.01	4.11	7.38	3.28	1.97	6.38	1.29
	3SD	9.84	9.84	9.84	5.50	0.55	7.87	21.31	26.23	3.69	1.30	0.33	0.00	0.66	8.00	0.77
	MAD	19.34	21.31	19.34	5.32	21.32	29.51	47.54	52.13	4.93	30.31	21.97	21.31	19.67	5.74	29.03
	REF INT	10.98	9.84	9.84	5.55	3.17	6.56	19.67	26.23	3.45	3.50	1.48	3.28	3.61	4.11	4.53
	BOOT	47.05	50.82	51.48	5.33	58.60	33.28	54.10	59.34	4.96	36.27	42.62	45.90	39.67	5.38	57.90
	JK	46.39	50.82	52.13	5.33	58.74	33.28	54.10	59.34	5.05	36.28	42.62	40.98	38.69	5.32	58.03
	MCMC HMC NUT	5.08	3.28	0.66	6.55	63.01	49.34	65.57	73.77	5.05	37.04	0.00	0.00	0.00	0.00	60.86
	MCMC MH	22.79	26.23	28.85	5.60	16.70	49.34	65.57	73.77	5.05	37.04	44.10	44.26	41.31	5.37	60.87

*Note:* Performance metrics for each benchmark selection method and each benchmark curve method for the finishing phase. MCMC HMC NUT, Markov chain Hamiltonian Monte Carlo. TTD, time-to-detect disease.

Abbreviations: Avg, average; BOOT, bootstrapping; DR, detection rate; EDR, early detection rate; JK, jackkinfe; LDR, late detection rate; MAD, median absolute deviation; MCMC MH, Markov chain Monte Carlo Metropolis-Hastings; Mort., mortality; REF INT, reference interval; SD, standard deviation.

## Data Availability

The data that support the findings of this study are available from the Hanor swine production system and Prairie Systems. Restrictions apply to the availability of these data, which were used under license for this study. The data are available from the authors with permission from Hanor swine production system and Prairie Systems.

## Supporting Information

Supporting Information An Excel file with two spreadsheets was provided as supporting information. The spreadsheet with the name “Supporting Information 1” shows the results of the benchmarking methods for nursery lots considering the different health challenges. For the spreadsheet “Supporting Information 2,” this spreadsheet shows the results of the benchmarking methods for finishing lots considering the different health challenges.
